# Supplementary material for: Quantitative analysis of C. elegans transcripts by Nanopore direct-cDNA sequencing reveals terminal hairpins in non trans-spliced mRNAs
Source: Nat Commun. 2023 Mar 3;14:1229. doi: 10.1038/s41467-023-36915-0 (PMC9984361; doi:10.1038/s41467-023-36915-0)
Supplement: Supplementary file 1 — Supplementary Information [file 41467_2023_36915_MOESM1_ESM.pdf]

SUPPLEMENTARY FIGURES

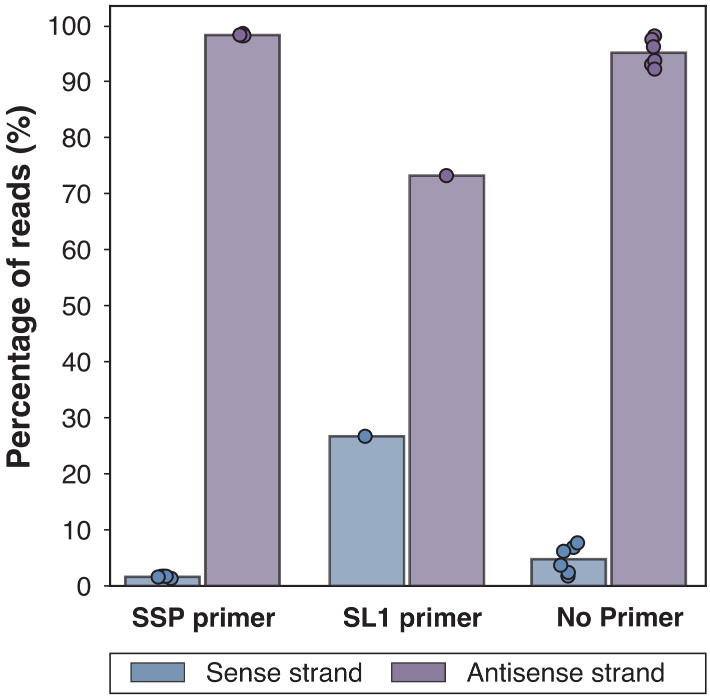

Supplementary Fig. 1: Strand bias in ONT direct-cDNA experiments. For each experiment, we measured the number of reads coming from each strand of the cDNA molecule sequenced. **SSP primer:** 2nd strand synthesis using the SSP primer (n=6). **SL1 primer:** 2nd strand synthesis using a SL1-specific primer (n=1). **No primer:** 2nd strand synthesis without using any primer (n=5).

Supplementary Fig. 2: a Schematic representation of expected cDNA structure. After ONT direct cDNA library preparation protocol double stranded cDNA is flanked by additional sequences that are expected to be recovered in the soft-clipped region of aligned reads. **b Measured length of 5' and 3' soft-clips.** Size distribution of observed soft-clips separated by read strands. 5' soft clip of antisense strand reads are in majority much larger than expected. **c Schematic representation of genomic read alignments obtained in *C. elegans* direct-cDNA experiments.** Purple region represent the region of a read mapped onto the genome. Soft-clip regions (in 5' and 3', per gene orientation) corresponds to unmapped part of the read.

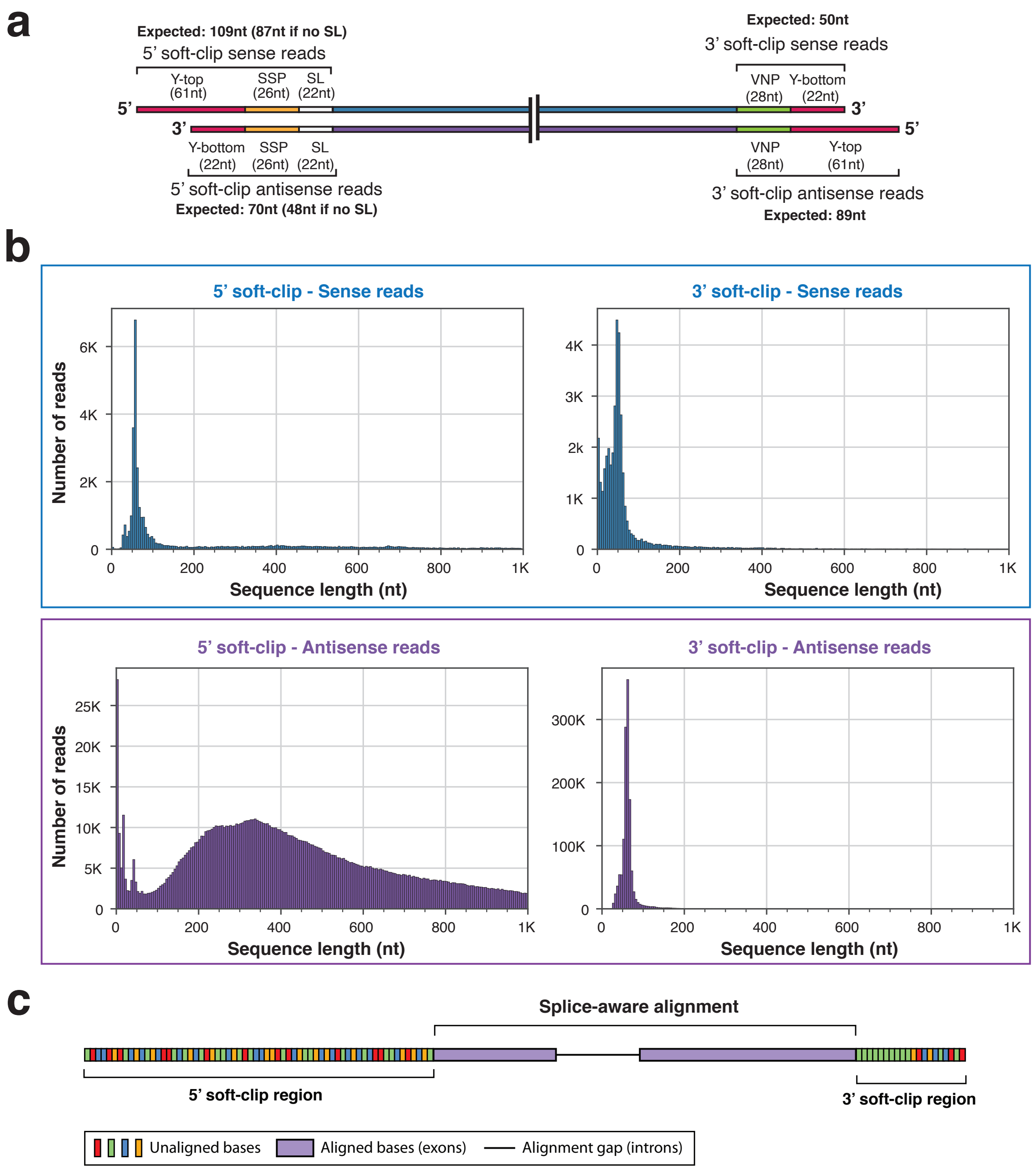
a
Expected: 109nt (87nt if no SL)
5' soft-clip sense reads
Y-top (61nt)
SSP (26nt)
SL (22nt)
5'
3'
Expected: 50nt
3' soft-clip sense reads
VNP (28nt)
Y-bottom (22nt)
3'
5'
Y-bottom (22nt)
SSP (26nt)
SL (22nt)
5' soft-clip antisense reads
Expected: 70nt (48nt if no SL)
VNP (28nt)
Y-top (61nt)
3' soft-clip antisense reads
Expected: 89nt
b
5' soft-clip - Sense reads
3' soft-clip - Sense reads
Number of reads
Sequence length (nt)
5' soft-clip - Antisense reads
3' soft-clip - Antisense reads
c
Splice-aware alignment
5' soft-clip region
3' soft-clip region
Unaligned bases
Aligned bases (exons)
Alignment gap (introns)

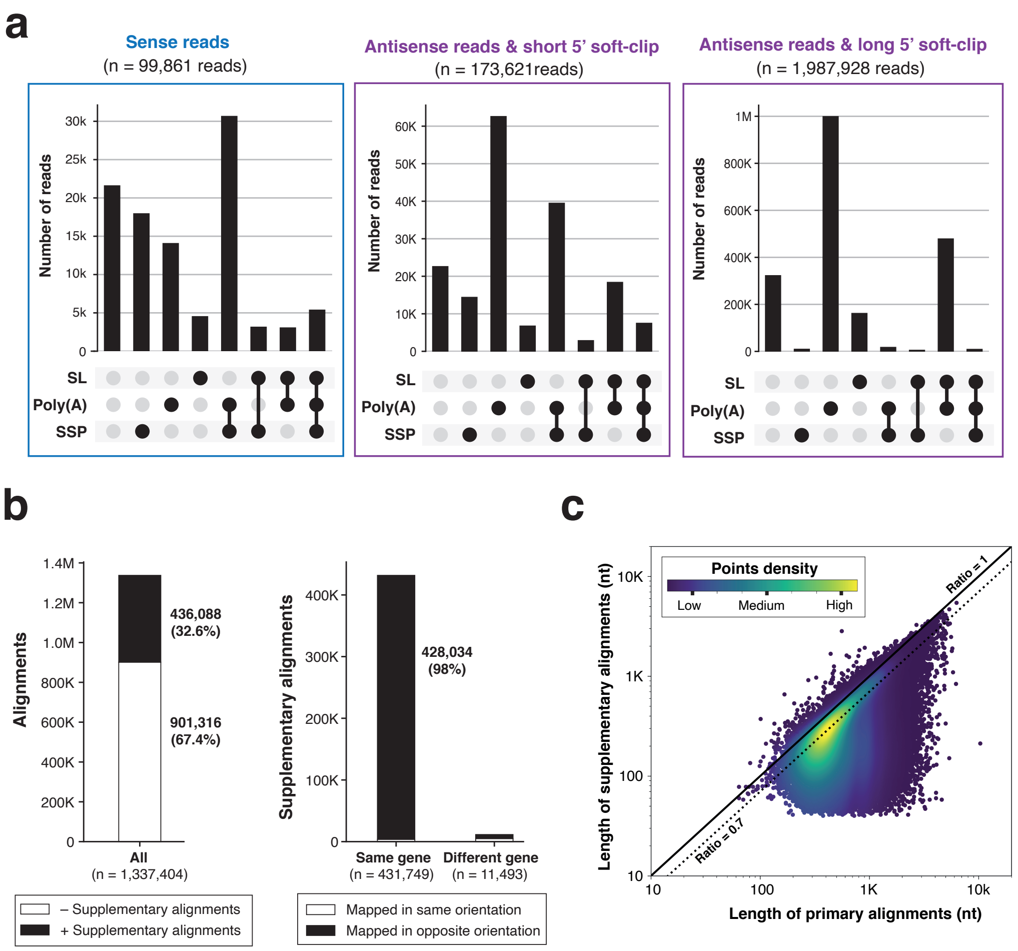

Supplementary Fig. 3: a: Quantification of sequencing adapters in soft-clips regions. For each read evaluated, we performed a sequence search for the SSP and SL motifs on the 5' soft-clip region, and for the poly(A) motif on the 3' soft-clip region **b: Origin of supplementary alignments.** 32.6% of aligned reads with with long 5' soft-clip have supplementary alignments. Among those, 98% are mapping to the same gene than the primary alignment, but in the opposite orientation. 32.6% of reads with primary alignments possess supplementary alignments. Among those, 98% are mapped to the same gene as in the primary alignment, but in the opposite orientation. **c: Size distribution of supplementary alignments.** Sizes of primary and supplementary alignments were compared for reads possessing both. Solid black line represent supplementary alignments that are the same size as their primary alignment (ratio 1/1). Dotted black line represent supplementary alignments that 70% of the size of their primary alignment (ratio 1/0.7).

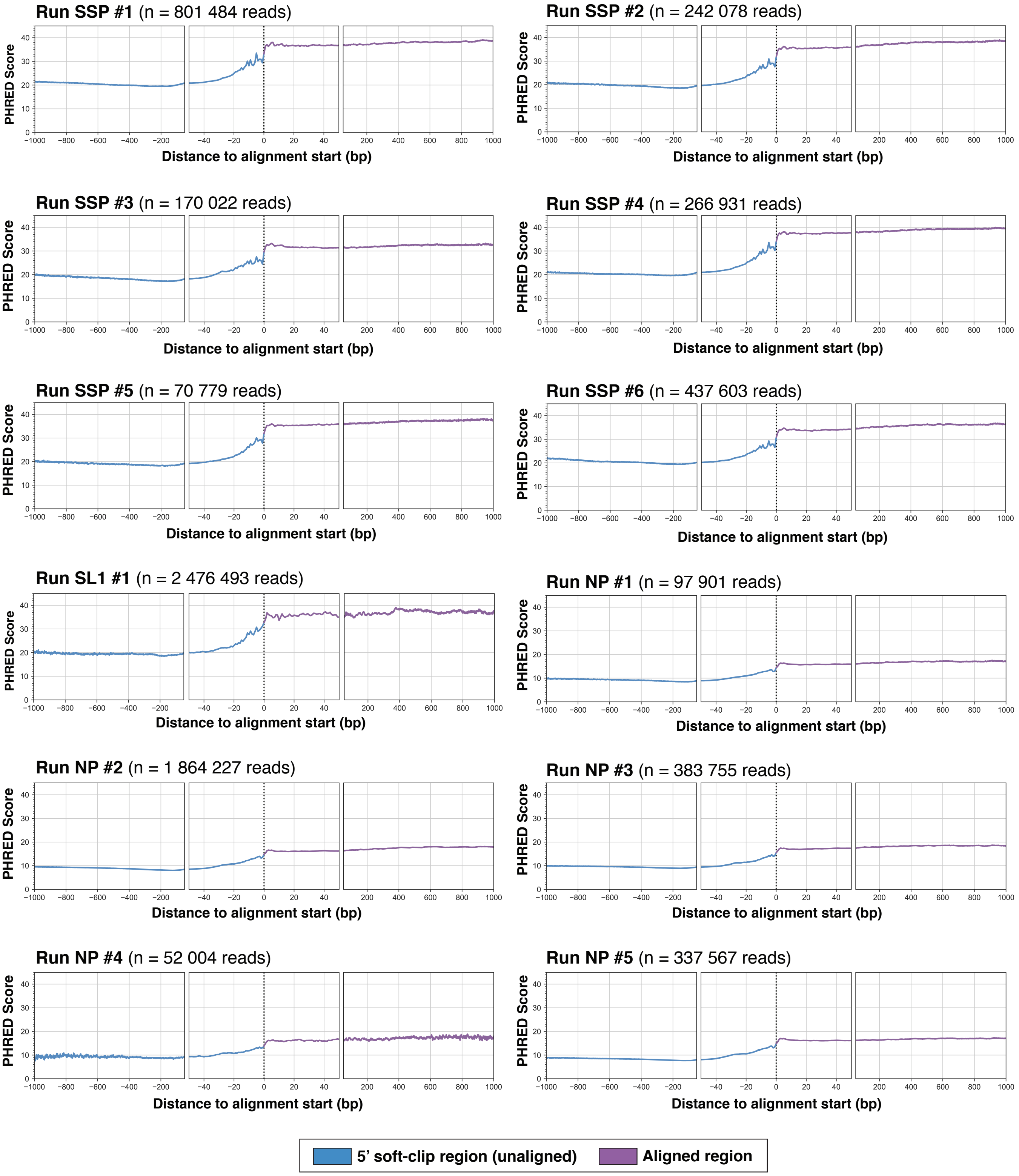

Supplementary Fig. 4: Base quality measures in each experiment. We measured the average base quality value over all the Nanopore reads obtained in each sequencing experiment. 5’ soft-clip region is represented in blue and aligned region (primary alignment) is represented in purple.

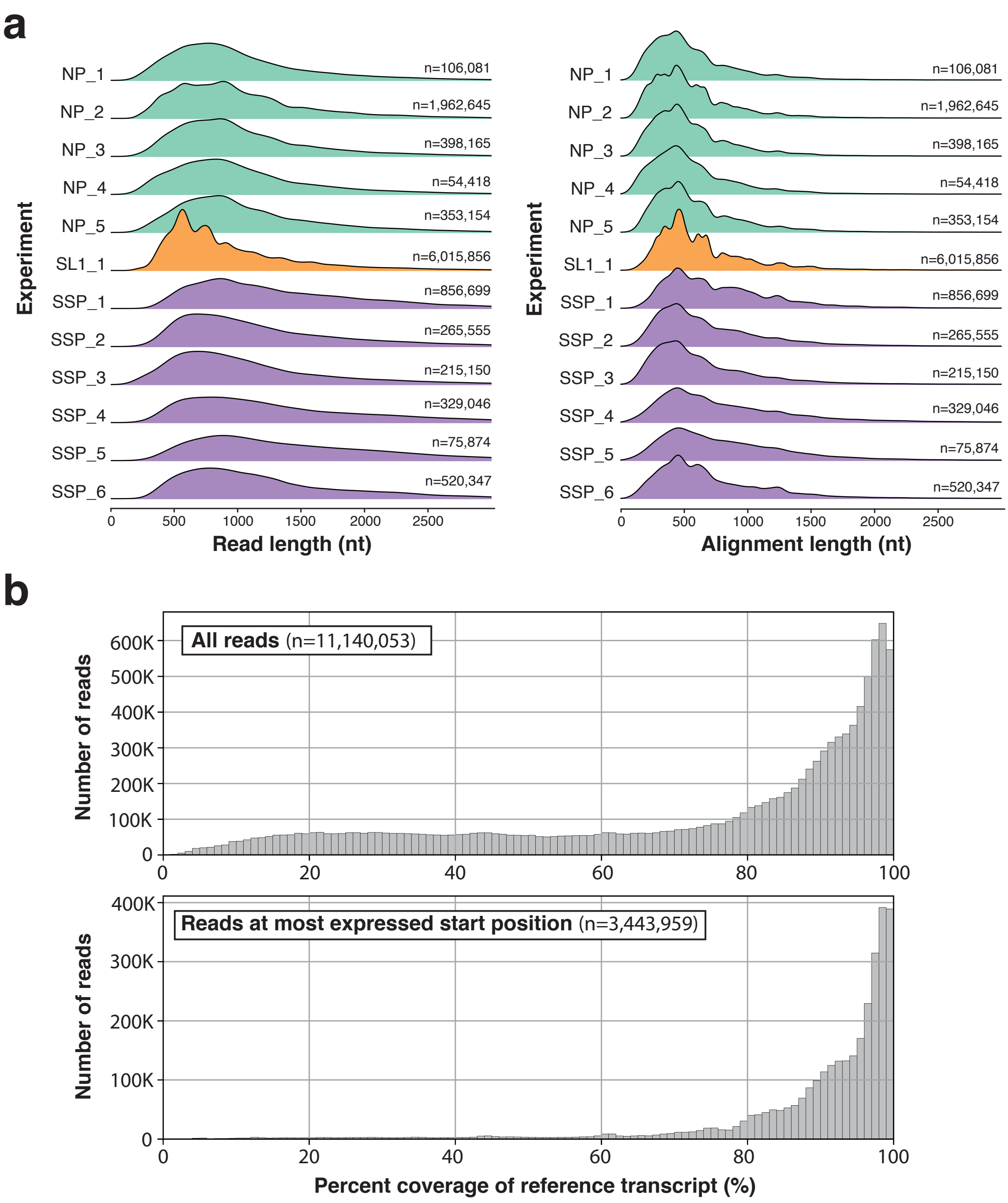

Supplementary Fig. 5: a: Read length and Alignment length are plotted as a histogram for each sequencing experiment and depicted as a joyplot. **b**: We measured their coverage of their respective reference transcript. The top Panel present the resulting distribution for all reads, the bottom one only for reads located at the most expressed position of each gene.

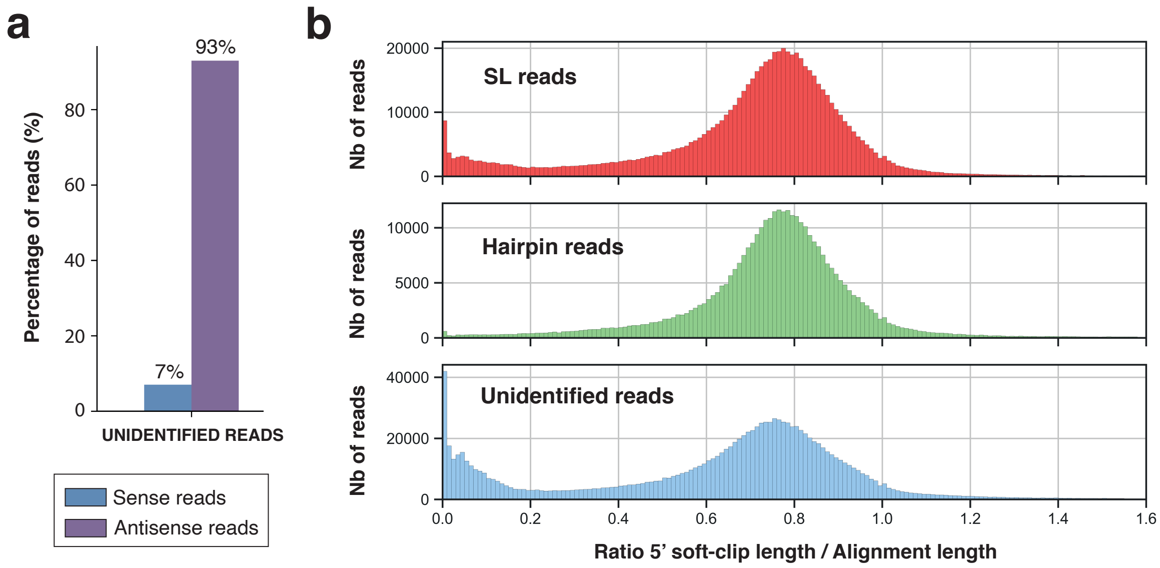

Supplementary Fig. 6: a: Strand orientation of reads for which we could not detect a SL sequence or an endogenous hairpin. **b**: Comparison of the length of 5' soft-clips versus the length of their alignment for reads considered SL (SL sequence found), Hairpin (Hairpin sequence found but no SL sequence) or unidentified (no SL or hairpin sequence found)

a

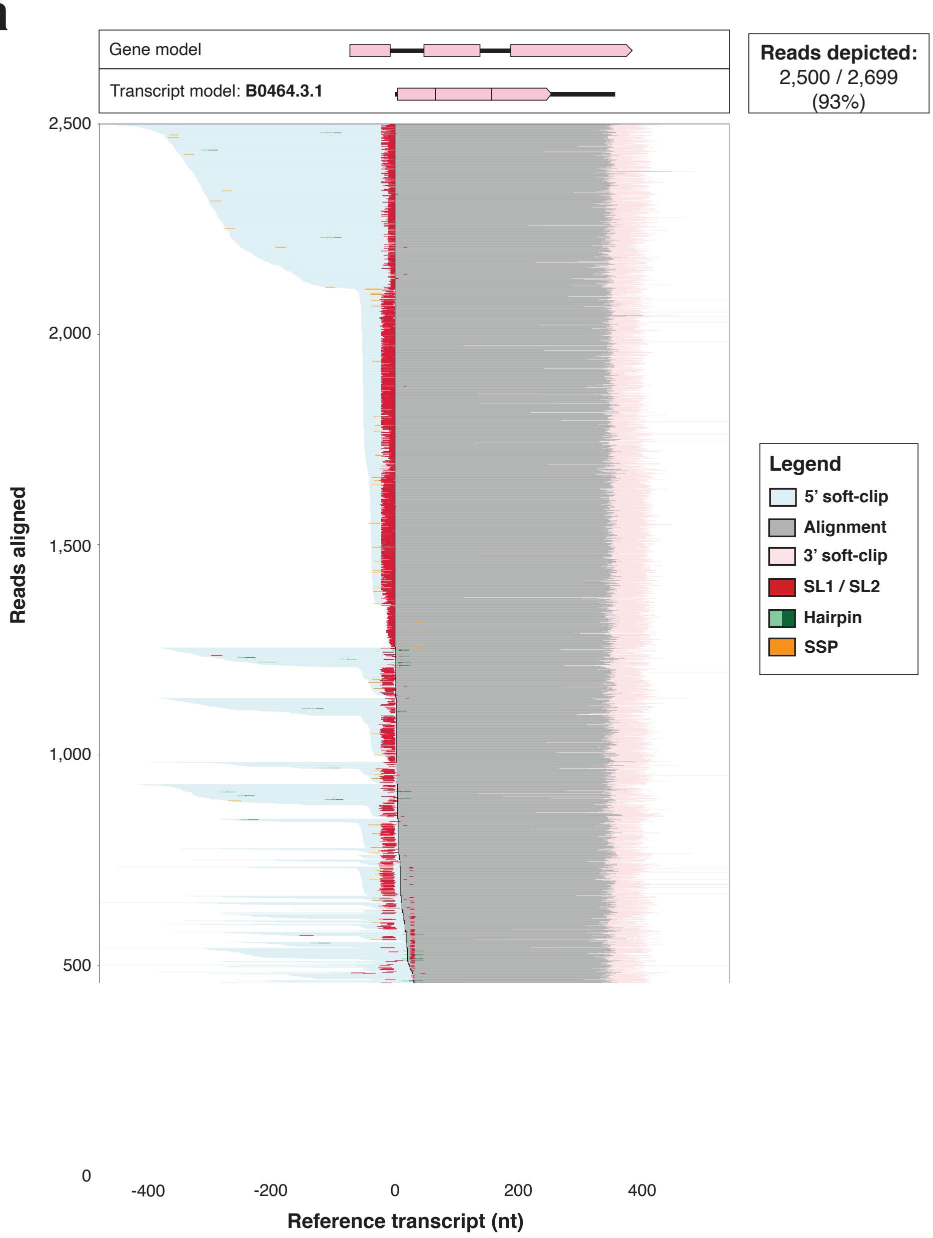

Supplementary Fig. 7a: Expanded representation of read features identified for each annotated gene isoform.

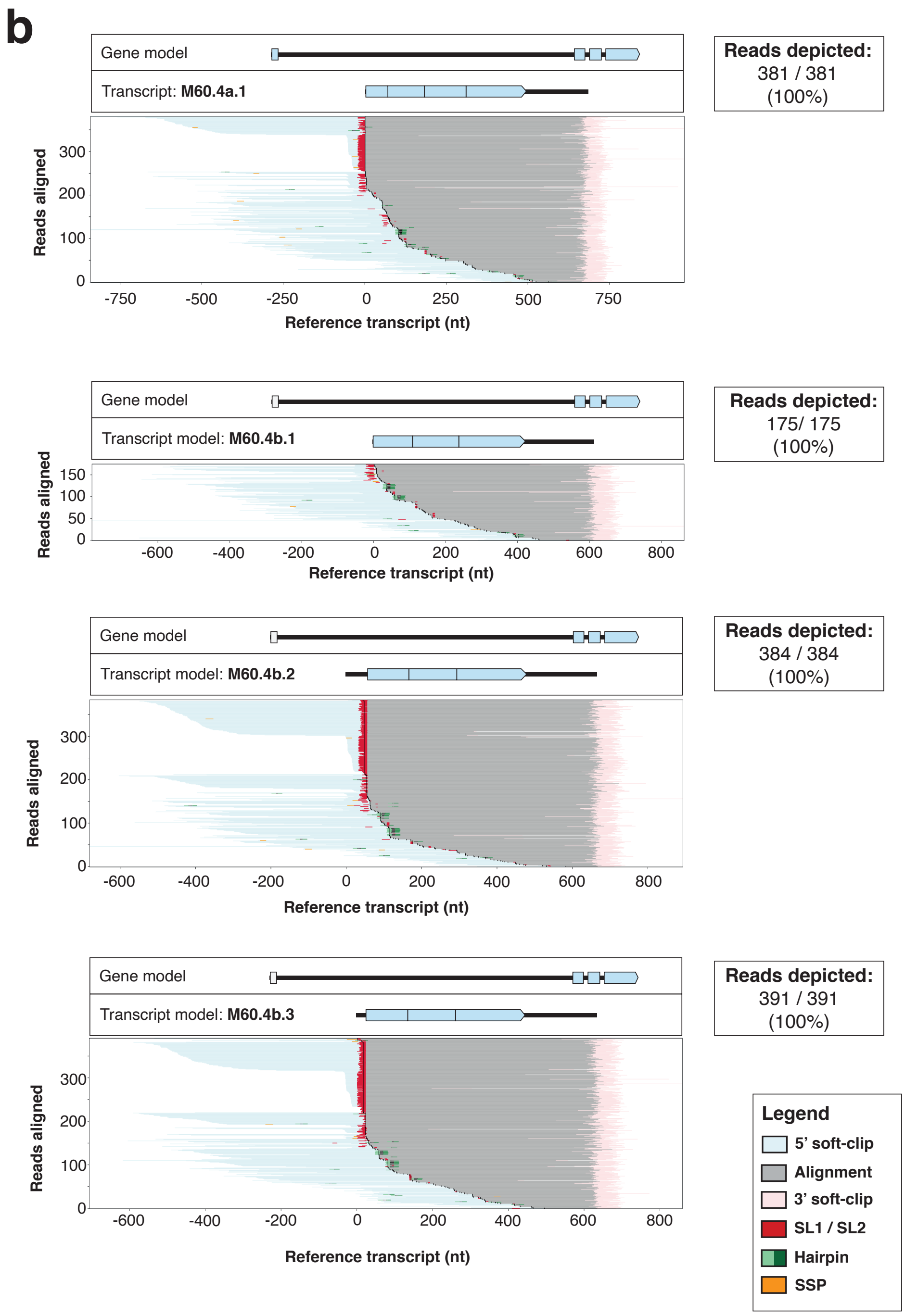

Supplementary Fig. 7b: Expanded representation of read features identified for each annotated gene isoform.

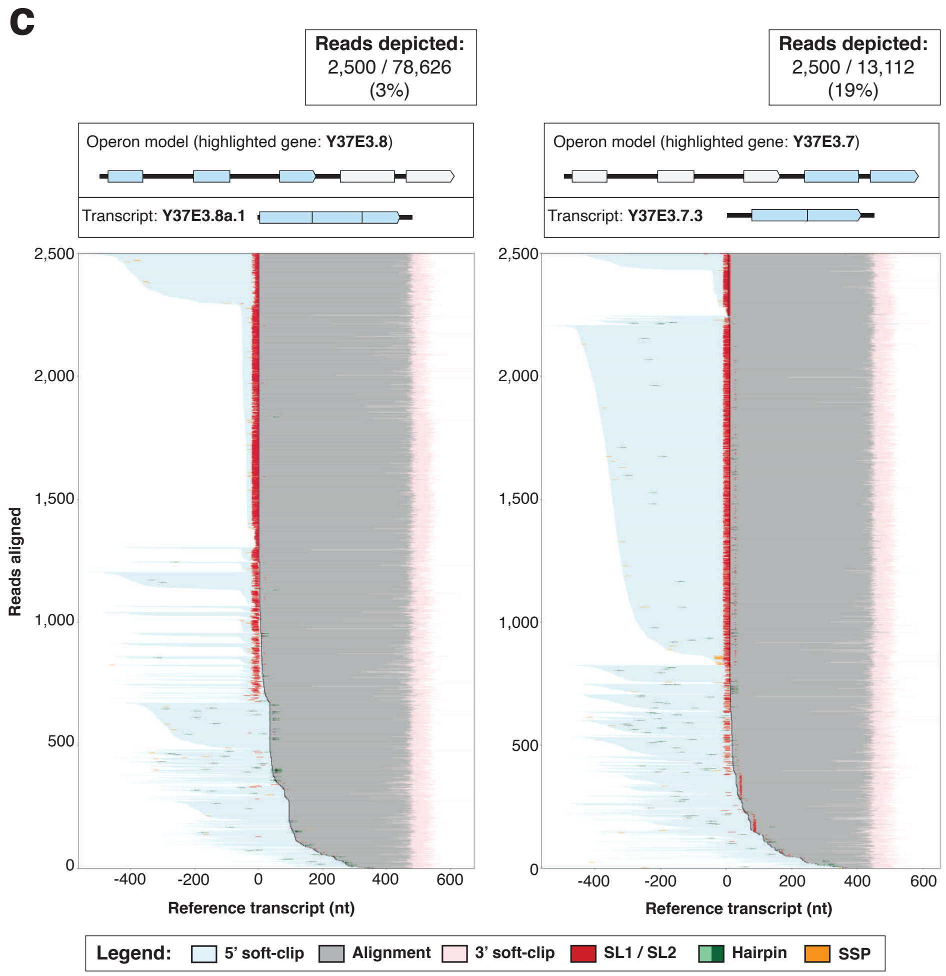

Supplementary Fig. 7c: Expanded representation of read features identified for each annotated gene isoform.

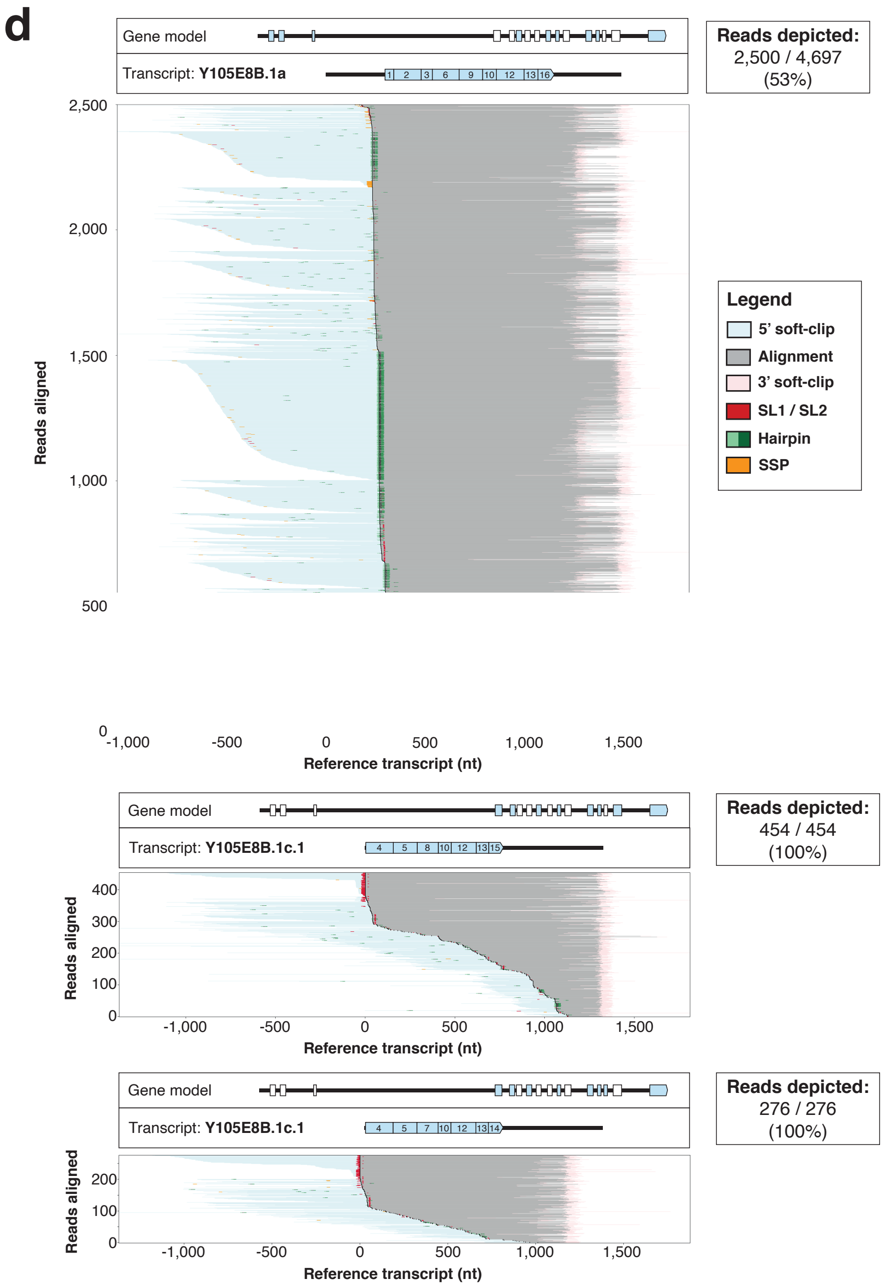

Supplementary Fig. 7d: Expanded representation of read features identified for each annotated gene isoform.

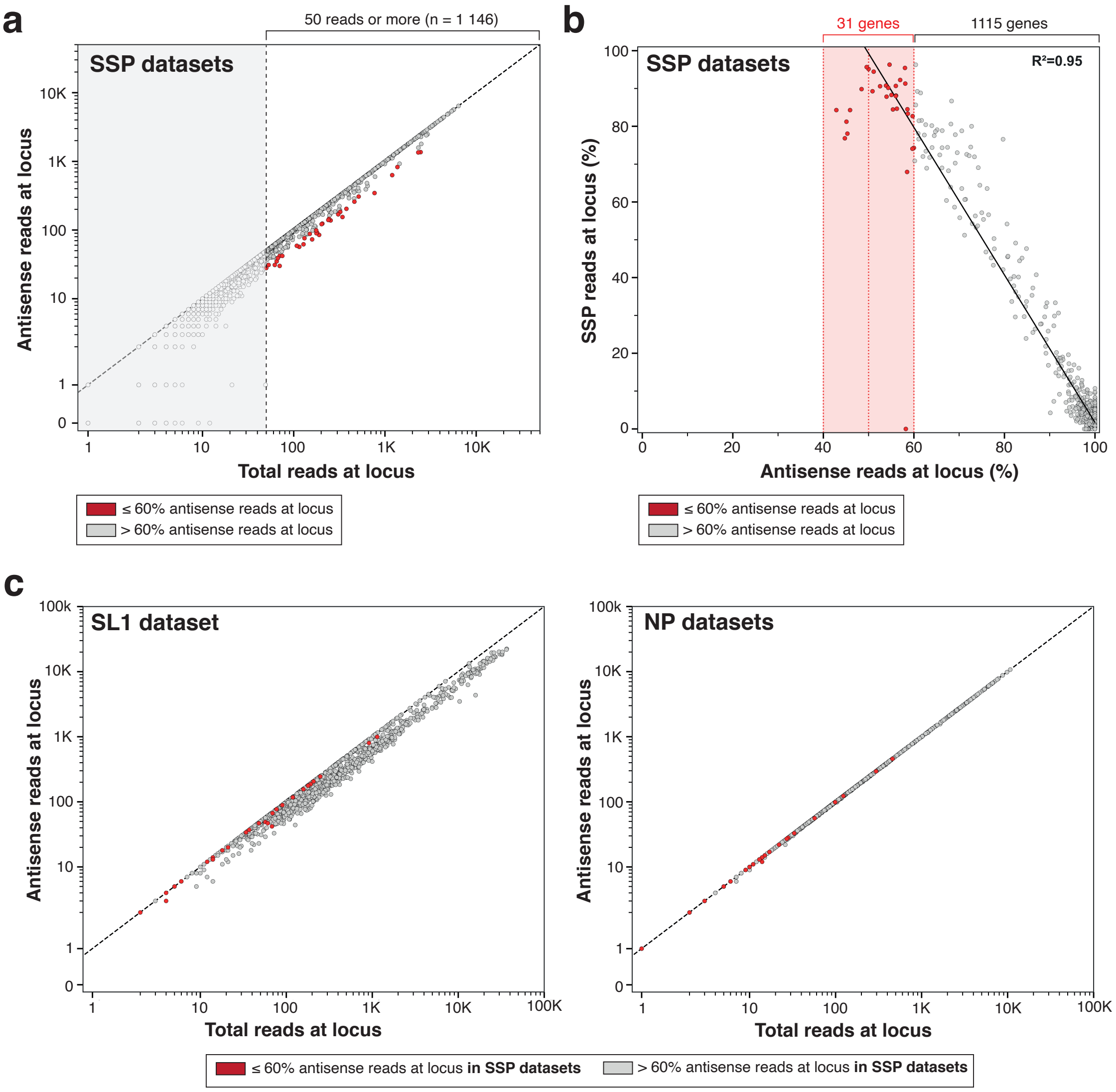

Supplementary Fig. 8: a: For each gene detected in the SSP dataset, we selected the most frequent 5' alignment start position and, if they had at least 50 reads, we plotted the number of antisense reads vs the total number of reads present at the position (n=1,146). The 31 genes displaying 60% or less antisense reads are highlighted in red. **b**: Linear regression analysis for the number of antisense reads (x-axis) and the percentage of reads for which we could detect the Strand Switching Primer (SSP) sequence (y-axis) in SSP datasets. **c**: For each locus, we plotted the total number of reads vs the number of antisense reads as shown in panel a considering only reads obtained in the SL1 dataset (left) and the NP datasets (right). As above, genes high proportion of antisense reads in the SSP datasets are shown in red.

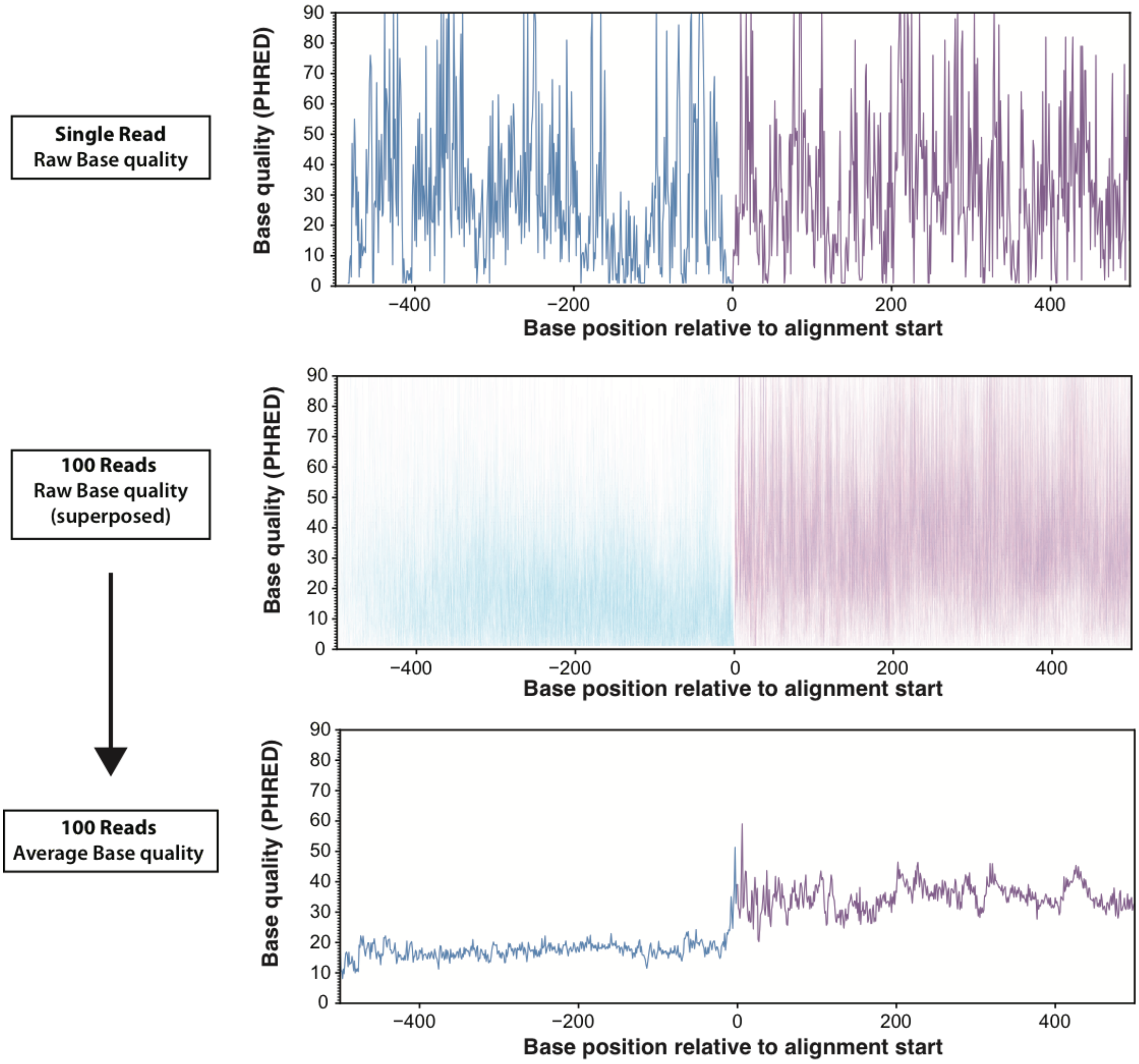

Supplementary Fig. 9: Method for evaluating base quality. Single read's base quality contains too much variability for accurate measurement (top panel). Trends in base quality can be observed when looking at larger samples of reads (middle panel - 100 reads shown together). Averaging the base quality per position across a large sample of reads (bottom panel) allows to accurately visualize differences in base quality between different regions of the reads. Unaligned 5' soft-clip region is shown in blue and alignment region in purple.

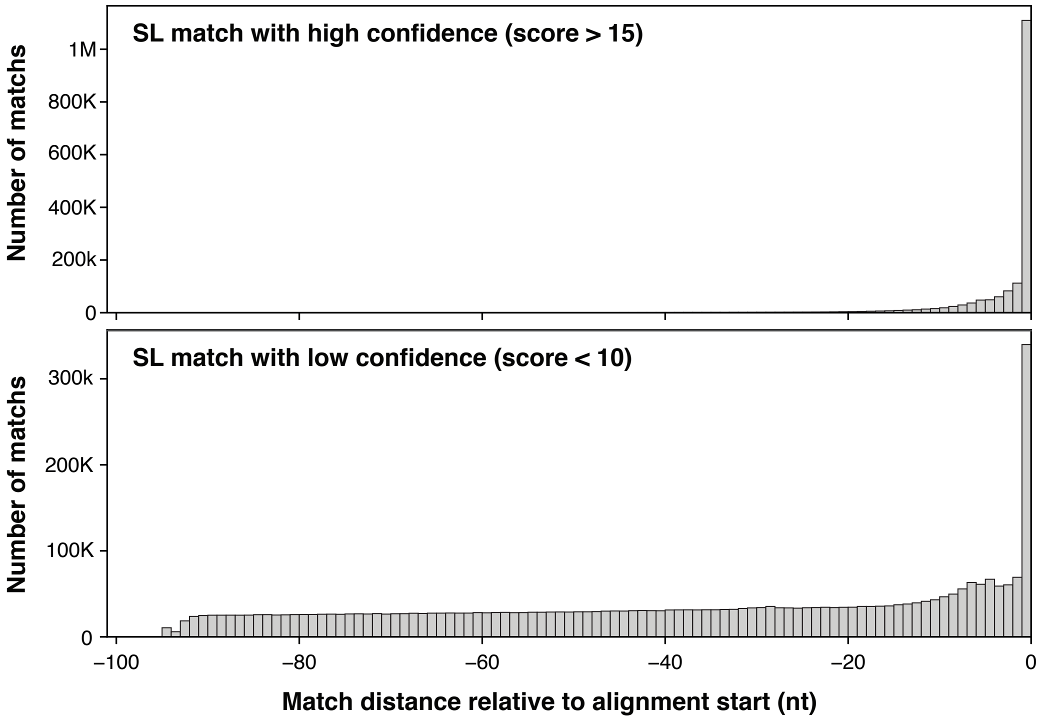

Supplementary Fig. 10: High confidence SL matches are preferentially located near the alignment start. For each SL sequence identified, we measured their distance to the alignment start. As expected, matches which scored high (top panel) are found in the direct vicinity of the alignment start. Low-scoring matches (bottom panel) are generally found all across the 5’ soft-clip region, but a sub-set of those are found where expected, making those more reliable.

TABLES

	Total Basecalled	mRNA		rRNA		other ncRNA		unmapped	
**SSP_1**	1,067,006	856,699	80.29%	26,155	2.45%	2,433	0.23%	181,719	17.03%
**SSP_2**	372,176	265,555	71.35%	24,802	6.66%	1,982	0.53%	79,837	21.45%
**SSP_3**	473,394	215,150	45.45%	31,680	6.69%	3,240	0.68%	223,324	47.18%
**SSP_4**	804,456	329,046	40.90%	164,953	20.50%	3,348	0.42%	307,109	38.18%
**SSP_5**	203,352	75,874	37.31%	28,633	14.08%	801	0.39%	98,044	48.21%
**SSP_6**	2,698,210	520,347	19.28%	1,346,202	49.89%	17,779	0.66%	813,882	30.16%
**SL1_1**	7,811,012	6,015,856	77.02%	541,476	6.93%	32,440	0.42%	1,221,240	15.63%
**NP_1**	330,271	106,081	32.12%	32,045	9.70%	1,413	0.43%	190,732	57.75%
**NP_2**	3,238,319	1,962,645	60.61%	593,668	18.33%	83,453	2.58%	598,553	18.48%
**NP_3**	630,506	398,165	63.15%	102,889	16.32%	7,333	1.16%	122,119	19.37%
**NP_4**	99,031	54,418	54.95%	19,225	19.41%	3,210	3.24%	22,178	22.40%
**NP_5**	575,200	353,154	61.40%	80,186	13.94%	7,714	1.34%	134,146	23.32%

Supplementary Table 1: Alignment statistics by biotype.

a

	Basecalled	PASS		FAIL	
		Total	Aligned	Total	Aligned
**SSP_1**	1,067,062	956,806	828,136	110,200	28,563
**SSP_2**	372,188	292,038	250,067	80,139	15,488
**SSP_3**	428,451	238,915	188,634	189,479	26,516
**SSP_4**	805,214	709,731	320,076	95,325	8,970
**SSP_5**	203,384	166,521	72,555	36,831	3,319
**SSP_6**	2,698,484	2,285,907	492,179	412,304	28,168
**SL1_1**	7,811,076	6,701,520	5,748,355	1,109,492	267,501
**NP_1**	330,272	219,458	78,430	110,813	27,651
**NP_2**	3,238,319	1,942,290	1,357,598	1,296,029	605,047
**NP_3**	630,506	430,424	325,555	200,082	72,610
**NP_4**	99,031	60,990	42,368	38,041	12,050
**NP_5**	575,203	287,458	213,590	287,742	139,564
**Total**	18,259,190	14,292,058	9,917,543	3,966,477	1,235,447

b

		SL		Hairpin		Unidentified	
	**Total**	Total	%	Total	%	Total	%
**PASS**	9,915,859	4,864,725	49%	1,122,274	11%	3,928,860	40%
**FAIL**	1,224,194	379,062	31%	141,949	10%	703,183	57%

Supplementary Table 2: **a :** Pass/Fail status of reads included in the analysis. **b:** Distribution of 5' features identified in Pass/ Fail reads

Sequencing Exp.	BioSample	Genomic alignments	Transcriptomic alignments
**SSP_1**	SAMN27178168	SRR18584063	SRR18688508
**SSP_2**		SRR18584064	SRR18688509
**SSP_3**		SRR18584067	SRR18688512
**SSP_4**		SRR18584068	SRR18688513
**SSP_5**		SRR18584069	SRR18688514
**SSP_6**		SRR18584070	SRR18688515
**SL1_1**	SAMN27178169	SRR18584059	SRR18688504
**NP_1**	SAMN27178170	SRR18584060	SRR18688505
**NP_2**		SRR18584061	SRR18688506
**NP_3**		SRR18584062	SRR18688507
**NP_4**		SRR18584065	SRR18688510
**NP_5**		SRR18584066	SRR18688511

Supplementary Table 3: Sequence Read Archive (SRA) codes for accessing datasets.
