# Supplementary material for: Quantitative analysis of C. elegans transcripts by Nanopore direct-cDNA sequencing reveals terminal hairpins in non trans-spliced mRNAs
Source: Nat Commun. 2023 Mar 3;14:1229. doi: 10.1038/s41467-023-36915-0 (PMC9984361; doi:10.1038/s41467-023-36915-0)
Supplement: Supplementary file 3 — Reporting Summary [file 41467_2023_36915_MOESM3_ESM.pdf]

Corresponding author(s): Denis Dupuy

Last updated by author(s): Dec 13, 2022

Reporting Summary

Nature Portfolio wishes to improve the reproducibility of the work that we publish. This form provides structure for consistency and transparency in reporting. For further information on Nature Portfolio policies, see our Editorial Policies and the Editorial Policy Checklist.

Statistics

For all statistical analyses, confirm that the following items are present in the figure legend, table legend, main text, or Methods section.

n/a	Confirmed	
☐	☒	The exact sample size (*n*) for each experimental group/condition, given as a discrete number and unit of measurement
☐	☒	A statement on whether measurements were taken from distinct samples or whether the same sample was measured repeatedly
☒	☐	The statistical test(s) used AND whether they are one- or two-sided<br>*Only common tests should be described solely by name; describe more complex techniques in the Methods section.*
☒	☐	A description of all covariates tested
☒	☐	A description of any assumptions or corrections, such as tests of normality and adjustment for multiple comparisons
☐	☒	A full description of the statistical parameters including central tendency (e.g. means) or other basic estimates (e.g. regression coefficient) AND variation (e.g. standard deviation) or associated estimates of uncertainty (e.g. confidence intervals)
☒	☐	For null hypothesis testing, the test statistic (e.g. *F*, *t*, *r*) with confidence intervals, effect sizes, degrees of freedom and *P* value noted<br>*Give P values as exact values whenever suitable.*
☒	☐	For Bayesian analysis, information on the choice of priors and Markov chain Monte Carlo settings
☒	☐	For hierarchical and complex designs, identification of the appropriate level for tests and full reporting of outcomes
☒	☐	Estimates of effect sizes (e.g. Cohen's *d*, Pearson's *r*), indicating how they were calculated

Our web collection on statistics for biologists contains articles on many of the points above.

Software and code

Policy information about availability of computer code

Data collection	The data presented was generated experimentally in our lab and not collected
Data analysis	Custom softwares are available on Github as indicated in the data availability section of the manuscript: https://github.com/florianbrnrd/elegans-trans-splicing.

For manuscripts utilizing custom algorithms or software that are central to the research but not yet described in published literature, software must be made available to editors and reviewers. We strongly encourage code deposition in a community repository (e.g. GitHub). See the Nature Portfolio guidelines for submitting code & software for further information.

Data

Policy information about availability of data

All manuscripts must include a data availability statement. This statement should provide the following information, where applicable:

- Accession codes, unique identifiers, or web links for publicly available datasets
- A description of any restrictions on data availability
- For clinical datasets or third party data, please ensure that the statement adheres to our policy

Data availability
The direct-cDNA datasets used in this study have been deposited in the Sequence Read Archive (SRA) under the accession code PRJNA822363 and datasets are listed in the Supplementary Table 2.

March 2021

The dataset table, generated by processing all of the obtained alignment files, and used in all computational analysis has been deposited on Figshare, along with all the other files generated as part of our analysis (DOI: 10.6084/m9.figshare.19131260).
A companion web-app, allowing the reader to view our results regarding any of the genes found in our sequencing experiments is directly available here: https://elegans-trans-splicing.streamlit.app

Human research participants

Policy information about studies involving human research participants and Sex and Gender in Research.

Reporting on sex and gender	not applicable
Population characteristics	not applicable
Recruitment	not applicable
Ethics oversight	not applicable

Note that full information on the approval of the study protocol must also be provided in the manuscript.

Field-specific reporting

Please select the one below that is the best fit for your research. If you are not sure, read the appropriate sections before making your selection.

☒ Life sciences ☐ Behavioural & social sciences ☐ Ecological, evolutionary & environmental sciences

For a reference copy of the document with all sections, see nature.com/documents/nr-reporting-summary-flat.pdf

Life sciences study design

All studies must disclose on these points even when the disclosure is negative.

Sample size	We performed sequencing runs until at least three million individual sequence reads were obtained in a given condition
Data exclusions	No data were excluded from the analysis
Replication	The number of individual sequencing runs for each condition are indicated in the manuscript. The cumulative number of reads obtained was our criteria to add additional replicate or not (6 SSP, 5 NP and 1 SL1) .
Randomization	Not applicable because we are not comparing distinct populations
Blinding	Not applicable because there was no group allocation involved in this study.

Reporting for specific materials, systems and methods

We require information from authors about some types of materials, experimental systems and methods used in many studies. Here, indicate whether each material, system or method listed is relevant to your study. If you are not sure if a list item applies to your research, read the appropriate section before selecting a response.

Materials & experimental systems

n/a	Involved in the study	
☒	☐	Antibodies
☒	☐	Eukaryotic cell lines
☒	☐	Palaeontology and archaeology
☐	☒	Animals and other organisms
☒	☐	Clinical data
☒	☐	Dual use research of concern

Methods

n/a	Involved in the study	
☒	☐	ChIP-seq
☒	☐	Flow cytometry
☒	☐	MRI-based neuroimaging

Animals and other research organisms

Policy information about studies involving animals; ARRIVE guidelines recommended for reporting animal research, and Sex and Gender in Research

Laboratory animals	C. elegans strain N2
Wild animals	The study did not involve wild animals
Reporting on sex	not applicable. We used hermaphrodite WT population in the present study
Field-collected samples	The study did not involve collected samples
Ethics oversight	Nematodes are not subjected to ethics committee approval in our country.

Note that full information on the approval of the study protocol must also be provided in the manuscript.
